# NKG2D and DNAM-1 activating receptors and their ligands in NK-T cell interactions: role in the NK cell-mediated negative regulation of T cell responses

**DOI:** 10.3389/fimmu.2012.00408

**Published:** 2013-01-09

**Authors:** Alessandra Zingoni, Michele Ardolino, Angela Santoni, Cristina Cerboni

**Affiliations:** ^1^Department of Molecular Medicine, Istituto Pasteur-Fondazione Cenci Bolognetti, “Sapienza” University of RomeRome, Italy; ^2^NeuromedPozzilli, Italy

**Keywords:** NKG2D ligands, DNAM-1 ligands, NK–T cell cross-talk, DNA damage response, cell proliferation

## Abstract

The negative regulation of adaptive immunity is relevant to maintain lymphocyte homeostasis. Several studies on natural killer (NK) cells have shown a previously unappreciated immunomodulatory role, as they can negatively regulate T cell-mediated immune responses by direct killing and by secretion of inhibitory cytokines. The molecular mechanisms of T cell suppression by NK cells, however, remained elusive. Only in the last few years has it become evident that, upon activation, human T cells express MICA–B, ULBP1–3, and PVR, ligands of the activating receptors NKG2D and DNAM-1, respectively. Their expression renders T cells targets of NK cell lysis, representing a new mechanism taking part to the negative regulation of T cell responses. Studies on the expression of NKG2D and DNAM-1 ligands on T cells have also contributed in understanding that the activation of ATM (ataxia-telangiectasia, mutated)/ATR (ATM/Rad3-related) kinases and the DNA damage response is a common pathway regulating the expression of activating ligands in different types of cells and under different conditions. The functional consequences of NKG2D and DNAM-1 ligand expression on activated T cells are discussed in the context of physiologic and pathologic processes such as infections, autoimmunity, and graft versus host disease.

Natural killer (NK) cells contribute to the suppression of T cell responses and to the maintenance of T lymphocyte homeostasis through the release of inhibitory cytokines, such as TGF-β and IL-10, which can inhibit dendritic cell (DC) maturation or T cell activation and functions, and/or through the direct elimination of antigen-presenting cells and activated T cells (Andoniu et al., 2008). The activating receptors expressed by NK cells and involved in the direct elimination of T cells remain still elusive although they have been recently focus of intense research. 2B4/CD244, LFA-1/D11a/CD18, NKp46/CD335, NKp30/CD337, KIR2DS1/CD158h, and in particular NKG2D/CD314 and DNAM-1/CD226 appear to play a prominent role.

## EXPRESSION OF NKG2D AND DNAM-1 LIGANDS ON T CELLS

### NKG2D AND ITS LIGANDS

NKG2D is an activating receptor expressed on NK cells, CD8^+^ T cells and γ/δ T cells that binds to several inducible self-proteins belonging to the MIC (MICA, MICB) and ULBP (ULBP1 to ULBP6) families in humans, and H60 (a–c), Rae (α-ε) and MULT1 in mice (Eagle and Trowsdale, 2007). NKG2D ligands (NKG2DLs) are mainly induced on the surface of transformed, infected or otherwise “stressed” cells, while the expression on healthy cells is low. However, increasing evidences show that NKG2DLs can be constitutively expressed or induced on normal hematopoietic cells including bone marrow cells (Nowbakht et al., 2005; Poggi et al., 2005; Spaggiari et al., 2006), mature DCs (Jinushi et al., 2003a,b; Andoniou et al., 2005; Schrama et al., 2006; Galazka et al., 2007; Qiao et al., 2008), monocytes and macrophages (Hamerman et al., 2004; Nowbakht et al., 2005; Nedvetzki et al., 2007; Kloss et al., 2008; Schulz et al., 2010), B cells (Nowbakht et al., 2005), and T cells. In general, NKG2DLs are not expressed by resting T lymphocytes, but their expression can be induced by different stimuli (**Table 1**). The first evidence of NKG2DL expression on T cells came from a study by Molinero et al. (2002) indicating that human T cells can express MICA in response to alloantigen and to CD3/CD28 cross-linking. Furthermore, also other NKG2DLs namely MICA, MICB, and ULBP1–3, but not ULBP4, are detected on both CD4^+^ and CD8^+^ T lymphocytes following stimulation with alloantigens, SEB superantigen, a specific antigenic peptide or upon PMA/ionomycin treatment (Cerboni et al., 2007a,^2009^). As a consequence, activated T cells become susceptible to autologous NK cell lysis, with an NKG2D/NKG2DL-dependent mechanism (Cerboni et al., 2007a). Nielsen et al. (2012) further demonstrated that NKG2D, LFA-1, and NKp46 are involved in NK cell degranulation triggered by activated autologous CD4^+^ T cells, with both subsets of human NK cells (CD56^dim^ and CD56^bright^) equally cytotoxic. Expression of NKG2DLs was described also on regulatory T cells (Treg) in response to *Mycobacterium tuberculosis* and NK-cell mediated lysis of Treg involves both NKG2D and NKp46 (Roy et al., 2008). Expression of NKG2DLs was also reported on activated murine T cells. H60 is up-regulated on T cells upon *in vitro* stimulation with ovalbumin and T cell blasts become susceptible to syngeneic NK cell killing (Rabinovich et al., 2003). Of note, an *in vivo* study showed that chronic antigenic stimulation of CD4^+^ T lymphocytes determined up-regulation of H60 and MULT1 ligands (Noval Rivas et al., 2010). NKG2DL expression was observed on thymocytes of BALB/c mice and was modulated during thymocyte development, suggesting a possible but yet undefined function in this process (Li et al., 2005). In support of these findings, a role for NK cell-mediated cytotoxicity during thymocyte development was demonstrated (Schott et al., 2003).

**Table 1:** Different stimuli implicated in the induction or up-regulation of NKG2D and DNAM-1 ligands on activated T cells.

	**Stimulus**	**NKG2DLs**	**DNAM1Ls**	**T cell type**	**Reference**
Human	anti-CD3 plus anti-CD28	MICA/B, ULBP1–3	PVR	T cells	Molinero et al. (2002), Nielsen et al. (2012)
	Superantigen, alloantigen, PMA/ionomycine, antigenic peptide	MICA/B, ULBP1–3		CD4^+^ and CD8^+^ T cells	Cerboni et al. (2007a), Cerboni et al. (2009)
	Anti-CD3 plus IL-2	MICA		CD8^+^ T cells	Maasho et al. (2005)
	Superantigen		PVR, Nectin-2	T cells	Ardolino et al. (2011)
	PHA		PVR	CD4^+^ T cells	Cella et al. (2010)
	Histone deacetylase inhibitors	MICA/B		Jurkat and activated T cells	Skov et al. (2005)
	Propionic acid	MICA/B		Jurkat and activated CD4^+^ T cells	Andresen et al. (2009)
	*M. tuberculosis*	ULBP-1		Treg	Roy et al. (2008)
	HIV	MICA, ULBP-1, 2,3		Jurkat and activated CD4^+^ T cells	Cerboni et al. (2007b), Ward et al. (2007)
	HIV		PVR	Activated CD4^+^ T cells	Matusali et al. (2012)
Mouse	mHA antigen	H60, MULT1		CD4^+^ T cells	Noval Rivas et al. (2010)
	ConA, PMA/ionomycine, ovalbumin	H60		T cells	Rabinovich et al. (2003)

### DNAM-1 AND ITS LIGANDS

DNAM-1/CD226 is an activating receptor belonging to the Ig superfamily and is constitutively expressed by most NK cells, T cells, macrophages, and DCs. DNAM-1 interacts with LFA-1, required for its functional activity on both NK and cytotoxic T cells (Shibuya et al., 1996). Ligands for DNAM-1 (DNAM1Ls) include Nectin-2/CD112 and PVR/CD155 belonging to the Nectin/Nectin-like family of adhesion molecules (Bottino et al., 2003; Tahara-Hanaoka et al., 2004). The activating effects of DNAM-1 can be counteracted by TIGIT, a recently identified inhibitory receptor binding to PVR, and expressed by T and NK cells (Stanietsky et al., 2009). DNAM1Ls are broadly distributed on hematopoietic, epithelial, and endothelial cells as well as on several tumors (Bottino et al., 2003; Pende et al., 2006; Tahara-Hanaoka et al., 2006). We have shown that PVR and Nectin-2 are induced on T cells in response to SEB stimulation at both the mRNA and protein levels, but only PVR can reach the cell surface (Ardolino et al., 2011). PVR expression on CD4^+^ T lymphocytes was also observed upon phytohemagglutinin (PHA) stimulation or co-engagement of CD3/CD28 molecules (Cella et al., 2010; Nielsen et al., 2012; **Table 1**). DNAM-1/PVR axis is involved in the NK cell-mediated lysis of allogeneic activated T cells (Ardolino et al., 2011), while in an autologous setting, NKG2D emerges as the dominant receptor (Rabinovich et al., 2003; Cerboni et al., 2007a; Nielsen et al., 2012).

### ROLE OF THE DDR IN THE REGULATION OF NKG2D AND DNAM-1 LIGANDS ON ACTIVATED T LYMPHOCYTES

NKG2DL expression is tightly regulated at various levels. During malignant transformation, cells undergo genotoxic or other forms of cellular stress with *de novo* expression of NKG2DLs. Gasser et al. (2005) demonstrated that murine and human NKG2DLs are up-regulated in fibroblasts by genotoxic stress and stalled DNA replication, conditions known to activate the DNA damage response (DDR) initiated by ATM (ataxia-telangiectasia, mutated) or ATR (ATM/Rad3-related) kinases. Studies aimed at investigating the signaling pathways leading to NKG2DL expression on antigen-activated T cells highlighted a role for the DDR as well. Treatment with ATM/ATR inhibitors blocked MICA induction on T cells with a mechanism involving NF-κB (Cerboni et al., 2007a), which regulates MICA expression on activated T lymphocytes by binding a specific sequence in the long intron 1 of the *MICA* gene (Molinero et al., 2004). NKG2DLs were found also on HIV-infected CD4^+^ T cells and their expression requires the activation of the DDR and stress pathways, via the HIV-1-encoded molecule Vpr (Ward et al., 2009; Richard et al., 2010; Pham et al., 2011), a potent activator of ATR and of a cell cycle arrest in G_2_ (He et al., 1995; Jowett et al., 1995; Re et al., 1995; Roshal et al., 2003). Similarly, oxidative stress and DDR strongly contribute to induce PVR expression on activated T cells (Ardolino et al., 2011). Thus, activation of ATM/ATR kinases and DDR could be a common pathway regulating the expression of different activating ligands on T lymphocytes. Of note, we also found that genotoxic stress triggered ATM/ATR-dependent up-regulation of both DNAM1Ls and NKG2DLs on multiple myeloma cells (Soriani et al., 2009).

**FIGURE 1:**
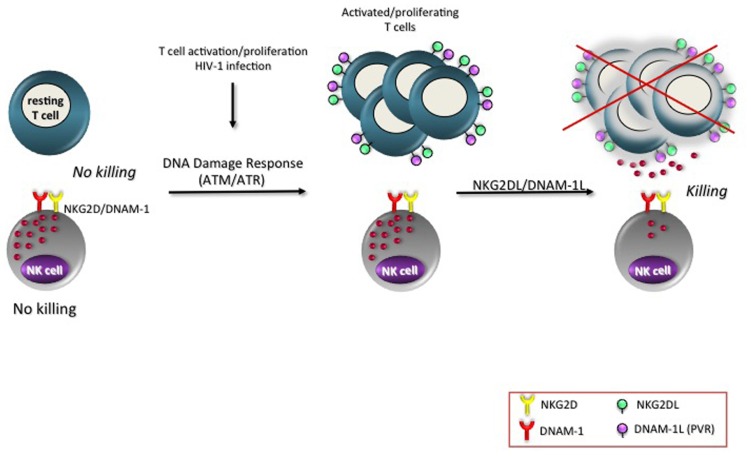
**NKG2D and DNAM-1 ligands are expressed on activated/proliferating T cells**. Resting T cells do not express ligands of the activating receptors NKG2D and DNAM-1 and are resistant to NK cell-mediated killing. However, upon T cell activation triggered by different stimuli (listed in **Table 1**), as well as upon HIV-1 infection, MICA/B, ULBP1–3, and PVR become detectable on the cell surface, preferentially on T lymphocytes that were undergone at least one cell division. The signaling pathways regulating the expression of NKG2D and DNAM-1 ligands involve the activation of ATM/ATR and of their substrates (e.g., phosphorylation of the histone H2AX). The final outcome is the direct elimination of activated/proliferating T lymphocytes by NK cells.

Increasing evidences show the involvement of DDR in many physiological processes, such as mitosis (Oricchio et al., 2006), insulin response (Yang and Kastan, 2000), V(D)J recombination (Chen et al., 2000) or after lipopolysaccharide stimulation in macrophages (Eissmann et al., 2010). In addition, up-regulation of ATM protein levels was observed in PBMCs (peripheral blood mononuclear cells) in response to mitogenic stimuli (Fukao et al., 1999). Increased phosphorylation of either ATM or one of its substrates, the histone H2AX, was described on T cells upon CD3 triggering, PHA or SEB stimulation (Cerboni et al., 2007a; Tanaka et al., 2007; Ardolino et al., 2011). Remarkably, PVR and NKG2DLs expression was mainly observed on T cells that had gone through at least one mitosis (Cerboni et al., 2007a, 2009; Ardolino et al., 2011). This is only one of the numerous examples showing a correlation of either NKG2DL or PVR expression with cell proliferation. In murine bone marrow grafts, Rae-1 was detected on donor proliferating hematopoietic cells in the spleen of the transplant recipients rather than on the long-term hematopoietic stem cells (Ogasawara et al., 2005). The presence of MIC molecules on rheumatoid arthritis synoviocytes was strongly associated with the expression of the nuclear Ki-67 proliferation marker (Groh et al., 2003) and *MIC* gene promoter contains elements for cell proliferation-associated transcriptional activation (Venkataraman et al., 2007). A preferential expression of PVR on proliferating rat hepatocytes during liver regeneration and acute injury was previously described (Erickson et al., 2006). These authors also reported that PVR expression in epithelial cells was tightly regulated by changes in cell density. NK cells react more efficiently to concanavalin A-stimulated, proliferating MHC class I-deficient target cells than to non-activated cells *in vitro* and *in vivo* (Correa et al., 1994) and proliferating T cells become more susceptible to NK cell killing (Ardolino et al., 2011). In line with these results, Davis’s group reported that human NK cells bound to cells in mitosis more effectively than the same cells in other phases of the cell cycle (Nolte-’t Hoen et al., 2007). Thus, we envisage that the expression of PVR and NKG2DLs on proliferating T lymphocytes is a possible mechanism used by NK cells to restrict the expansion of activated/proliferating T cells (**figure 1**).

## *IN VIVO* RELEVANCE FOR NK–T CELL INTERACTION

### VIRAL INFECTIONS

A plethora of studies analyzed the role of NKG2D/NKG2DL axis by looking at infected cells, which very often express one or more ligands. These studies have demonstrated that NKG2D plays an important role in anti-viral immunity, via a direct NK cell-mediated lysis of infected cells. This evidence is also underscored by the countermeasures taken by viruses to avoid NKG2D-mediated triggering (Lanier, 2008; Rossini et al., 2012). However, NK cell contribution to anti-viral immunity can be seen also from another point of view: NK cells might restrain anti-viral T cell responses, thus promoting the return to T cell homeostasis.

*In vivo* depletion studies established that NK cells act to negatively regulate CD4^+^ and CD8^+^ T cell-dependent IFN-γ production and proliferation during murine cytomegalovirus infection, and they can mediate a similar effect on CD4^+^ T cell responses during lymphocytic choriomeningitis virus (LCMV) infection in β2-microglobulin deficient mice (Su et al., 2001). Accordingly, a more recent study showed that perforin-deficient mice chronically infected with LCMV contain greater numbers of activated anti-viral T cells compared to control animals. The accumulation of activated CD8^+^ T cells resulted in mortality within 2–4 weeks, an event which is rarely seen following an i.p. injection with LCMV of normal mice (Matloubian et al., 1999). It was described also a three-way of NK–T cell interaction, where NK cells directly eliminate activated CD4^+^ T cells (via a perforin-dependent pathway), thereby affecting CD8^+^ T cell function with beneficial or detrimental effects depending on the viral dose. However, no role for NKG2D/NKG2DLs could be observed (Waggoner et al., 2011). In another study, NK cell depletion promoted LCMV-induced CD8^+^ T cell immunity with the involvement of both perforin and NKG2D (Lang et al., 2012). Altogether, these studies show that NK cells can be crucial in controlling viral infections not only by a direct elimination of infected cells, but also by altering the number and functions of virus-specific T cells. However, the role of NKG2D and other activating receptors awaits a better elucidation.

Considering HIV-1, a virus replicating (among other cell types) in CD4^+^ T lymphocytes, we face a situation where NK cell targeting of activated T cells via NKG2D means, at the same time, eliminating the infected cell. In fact, expression of several NKG2DLs was observed on HIV-1 infected CD4^+^ T cells, with increased susceptibility to NK lysis (Cerboni et al., 2007b; Ward et al., 2007; Fogli et al., 2008; Richard et al., 2010). However, HIV-1 has also evolved its own countermeasures as it can also down-regulate NKG2DLs via Nef and Vif proteins (Cerboni et al., 2007b; Norman et al., 2011). Thus, regulation of NKG2DL expression by Vpr, Nef and possibly other viral proteins might have different impacts on NK cell recognition of infected CD4^+^ T cells.

The role of DNAM-1 and its ligands in the context of NK–T cell interactions during viral infections has been less investigated. Recently, PVR was detected on HIV-1 infected CD4^+^ T cells, and when the NKG2D pathway was inhibited, additional blocking of DNAM-1 strongly impaired the capacity of NK cells to kill HIV-1-infected cells, indicating the involvement of both receptors (Matusali et al., 2012). However, expression of PVR on CD4^+^ T cells might also be responsible for the down-regulation of DNAM-1 on CD8^+^ T cells observed in chronic HIV-1 infection (Cella et al., 2010).

### AUTOIMMUNITY

The mechanisms by which NK cells modulate adaptive immune responses in the course of autoimmune diseases have been addressed by a large number of *in vivo* and *in vitro* studies. However, depending on the model system, NK cells might either promote or inhibit the generation and proliferation of autoreactive T cells (French and Yokoyama, 2004; Shi and Van Kaer, 2006; Flodström-Tullberg et al., 2009; Lünemann et al., 2009).

Thinking in terms of negative regulation of (autoreactive) T cell responses, NK cells might exert a direct effect on activated, autoantigen-specific T cells. In experimental autoimmune encephalomyelitis (EAE), *in vivo* depletion of NK cells exacerbated demyelination and the clinical features of EAE; in addition, *in vitro* studies have shown that direct NK–T cell contact inhibited T cell proliferation and cytokine production triggered by myelin-derived peptides (Zhang et al., 1997; Matsumoto et al., 1998; Smeltz et al., 1999; Xu et al., 2005). NK cells might thus ameliorate the course of EAE by limiting the expansion of myelin-reactive T cells in the periphery and in the absence of their suppressive action, central nervous system inflammation became more marked. However, these studies are in conflict with another report showing that NK cell depletion resulted in less severe clinical scores (Shi et al., 2000). In an *in vivo* model of colitis, NK cell-depleted animals developed accelerated disease, and it was suggested that NK cells inhibited effector CD4^+^ T cells in a perforin-dependent manner (Fort et al., 1998; Yamaji et al., 2012). Such a protective effect also occurred in *Staphylococcus aureus*- and collagen-induced arthritis (CIA; Nilsson et al., 1999; Leavenworth et al., 2011), as well as in NOD mice (Lee et al., 2004).

A number of studies have identified the cytolytic mechanism underlying NK cell-mediated killing of autoreactive T cells, and the NK cell-mediated immunoregulatory activity was shown to be perforin-dependent in animal models of colitis, EAE, and CIA (Fort et al., 1998; Lu et al., 2007; Leavenworth et al., 2011). Thus, the receptor/ligand interactions triggering a perforin-mediated cytotoxicity play a key role in controlling T cell responses, and NKG2D might be part of the picture, since it plays a major role in NK cell lysis of autologous activated T cells (Rabinovich et al., 2003; Cerboni et al., 2007a; Nielsen et al., 2012). Moreover, NK cells can lyse autologous DCs, that under certain circumstances – including EAE – express NKG2DLs (Jinushi et al., 2003a,b; Andoniou et al., 2005; Schrama et al., 2006; Galazka et al., 2007; Qiao et al., 2008). These data, together with NKG2DL expression also on activated macrophages and monocytes, bone marrow cells and microglia (Lünemann et al., 2008), indicate that this receptor/ligand pair might play a more general immunoregulatory role besides killing autoreactive T cells, e.g., by eliminating macrophages and other antigen-presenting cells or their precursors under inflammatory conditions, as a feedback mechanism to silence uncontrolled antigen-specific immune responses.

The role of NKG2D/NKG2DLs in autoimmunity has been addressed also from another point of view. In fact, endogenous cells and/or tissues can aberrantly express NKG2DLs (as shown in particular for MICA and MICB in humans and Rae-1 in mice), promoting activation of autoreactive infiltrating NKG2D^+^ T cells, leading to tissue destruction. Examples of this condition can be found in human type I diabetes and in NOD mice, in patients with rheumatoid arthritis, Crohn’s disease, celiac disease, and in a mouse model of autoimmune vitiligo. These aspects are however reviewed elsewhere (Shi and Van Kaer, 2006; Van Belle and von Herrath, 2009).

Regarding DNAM-1, despite the expression of PVR on activated T cells (Cella et al., 2010; Ardolino et al., 2011; Nielsen et al., 2012), its role was not evident in autologous NK–T combinations (Ardolino et al., 2011; Nielsen et al., 2012), while it was relevant in allogeneic settings (Ardolino et al., 2011), suggesting that DNAM-1 might not be involved in autoimmune reactions.

### GRAFT VERSUS HOST DISEASE

Allogeneic bone marrow transplantation (BMT) was estimated to be an effective treatment for hematologic malignancies and some solid tumors. However, the high incidence of graft versus host disease (GVHD) mediated by the activation and proliferation of alloreactive T cells leads to severe host tissue damage. Previous studies demonstrated that donor NK cells are able to suppress the development of GVHD through the killing of host antigen-presenting cells which are essential for donor T cell activation (Ruggeri et al., 2002). More recently, several *in vivo* studies in the mouse showed a direct effect of donor NK cells on GVHD-inducing T cells. Allogeneic T cells and NK cells trafficked similarly after BMT (Olson et al., 2009) and donor NK cells limited the expansion of syngeneic donor T cells through different mechanisms mediated by perforin and Fas-FasL interaction (Olson et al., 2010). Similarly, in a model of chronic GVHD, donor NK cells could restrain the expansion of antigen-specific CD4^+^ T cells responsible for GVHD. Interestingly, T cells under chronic antigenic stimulation up-regulated NKG2DLs and the immunoregulatory activity of NK cells was inhibited by injection of antibodies directed to NKG2D (Noval Rivas et al., 2010). In humans, a fraction of KIR2DS1^+^ NK cells can mediate strong alloreactivity against both mDCs and activated T lymphocytes and DNAM-1 and NKp30 were shown to be involved in this process, supporting an important role of NK cells in the prevention of GVHD (Sivori et al., 2011).

## CONCLUSION

In summary, a central role for NK cell killing in mediating immunoregulatory effects is emerging. Studies of different conditions (infections, autoimmunity, transplants) indicate that NK cytotoxicity of activated T cells, as well as of other cells of the immune system, is important in immune regulation. The involvement of NKG2D and DNAM-1 receptors may represent the “tip of the iceberg,” with significant effects on the negative regulation of adaptive T cell responses, resulting in increased viral burdens, viral persistence, and/or inflammation. The locations where these events take place, and which are the NK cell subsets involved are still rather obscure parts of the picture.

## Conflict of Interest Statement

The authors declare that the research was conducted in the absence of any commercial or financial relationships that could be construed as a potential conflict of interest.

## References

[B1] Andoniou C. E. van Dommelen S. L. Voigt V. Andrews D. M. Brizard G. Asselin-Paturel C. (2005). Interaction between conventional dendritic cells and natural killer cells is integral to the activation of effective antiviral immunity. *Nat. Immunol.* 10 1011–1019 16142239 10.1038/ni1244

[B2] Andoniu C. E. Coudert J. D Degli Esposti M. A. (2008). Killers and beyond: NK-cell-mediated control of immune response. *Eur. J. Immunol.* 38 2938–2942 18979519 10.1002/eji.200838882

[B3] Andresen L. Hansen K. A. Jensen H. Pedersen S. F. Stougaard P. Hansen H. R. (2009). Propionic acid secreted from propionibacteria induces NKG2D ligand expression on human-activated T lymphocytes and cancer cells. *J. Immunol.* 183 897–906 19553547 10.4049/jimmunol.0803014

[B4] Ardolino M. Zingoni A. Cerboni C. Cecere F. Soriani A. Iannitto M. L. (2011). DNAM-1 ligand expression on Ag-stimulated T lymphocytes is mediated by ROS-dependent activation of DNA-damage response: relevance for NK–T cell interaction. *Blood* 117 4778–4786 21406724 10.1182/blood-2010-08-300954

[B5] Bottino C. Castriconi R. Pende D. Rivera P. Nanni M. Carnemolla B. (2003). Identification of PVR (CD155) and Nectin-2 (CD112) as cell surface ligands for the human DNAM-1 (CD226) activating molecule. *J. Exp. Med.* 198 557–567 12913096 10.1084/jem.20030788 PMC2194180

[B6] Cella M. Presti R. Vermi W. Lavender K. Turnbull E. Ochsenbauer-Jambor C. (2010). Loss of DNAM-1 contributes to CD8^+^ T-cell exhaustion in chronic HIV-1 infection. *Eur. J. Immunol.* 40 949–954 20201043 10.1002/eji.200940234 PMC3031090

[B7] Cerboni C. Zingoni A. Cippitelli M. Piccoli M. Frati L. Santoni A. (2007a). Antigen-activated human T lymphocytes express cell-surface NKG2D ligands via an ATM/ATR-dependent mechanism and become susceptible to autologous NK-cell lysis. *Blood* 110 606–615 17405908 10.1182/blood-2006-10-052720

[B8] Cerboni C. Neri F. Casartelli N. Zingoni A. Cosman D. Rossi P. (2007b). Human immunodeficiency virus 1 Nef protein downmodulates the ligands of the activating receptor NKG2D and inhibits natural killer cell-mediated cytotoxicity. *J. Gen. Virol.* 88 242–250 17170457 10.1099/vir.0.82125-0

[B9] Cerboni C. Ardolino M. Santoni A. Zingoni A. (2009). Detuning CD8^+^ T lymphocytes by down-regulation of the activating receptor NKG2D: role of NKG2D ligands released by activated T cells. *Blood* 113 2955–2964 19124832 10.1182/blood-2008-06-165944

[B10] Chen H. T. Bhandoola A. Difilippantonio M. J. Zhu J. Brown M. J. Tai X. (2000). Response to RAG-mediated V(D)J cleavage by NBS1 and γ -H2AX. *Science* 290 1962–1965 11110662 10.1126/science.290.5498.1962 PMC4721589

[B11] Correa I. Corral L. Raulet D. H. (1994). Multiple natural killer cell-activating signals are inhibited by major histocompatibility complex class I expression in target cells. *Eur. J. Immunol.* 24 1323–1331 8206092 10.1002/eji.1830240613

[B12] Eagle R. A. Trowsdale J. (2007). Promiscuity and the single receptor: NKG2D. *Nat. Rev. Immunol.* 7 737–744 17673918 10.1038/nri2144

[B13] Eissmann P. Evans J. H. Mehrabi M. Rose E. L. Nedvetzki S. Davis D. M. (2010). Multiple mechanisms downstream of TLR-4 stimulation allow expression of NKG2D ligands to facilitate macrophage/NK cell crosstalk. *J. Immunol.* 184 6901–6909 20488792 10.4049/jimmunol.0903985

[B14] Erickson B. M. Thompson N. L. Hixson D. C. (2006). Tightly regulated induction of the adhesion molecule necl-5/CD155 during rat liver regeneration and acute liver injury. *Hepatology* 43 325–334 16440345 10.1002/hep.21021

[B15] Flodström-Tullberg M. Bryceson Y. T. Shi F. D. Höglund P. Ljunggren H. G. (2009). Natural killer cells in human autoimmunity. *Curr. Opin. Immunol.* 21 634–640 19892538 10.1016/j.coi.2009.09.012

[B16] Fogli M. Mavilio D. Brunetta E. Varchetta S. Ata K. Roby G. (2008). Lysis of endogenously infected CD4+ T cell blasts by rIL-2 activated autologous natural killer cells from HIV-infected viremic individuals. *PLoS Pathog.* 4:7 10.1371/journal.ppat.1000101. PMC2438610 18617991

[B17] Fort M. M. Leach M. W. Rennick D. M. (1998). A role for NK cells as regulators of CD4+ T cells in a transfer model of colitis. *J. Immunol.* 161 3256–3261 9759840

[B18] French A. R. Yokoyama W. M. (2004). Natural killer cells and autoimmunity. *Arthritis Res. Ther.* 6 8–14 14979926 10.1186/ar1034 PMC400423

[B19] Fukao T. Kaneko H. Birrell G. Tashita H. Yoshida T. Cross S. (1999). ATM is upregulated during the mitogenic response in peripheral blood mononuclear cells. *Blood* 94 1998–2006 10477729

[B20] Galazka G. Jurewicz A. Orlowski W. Stasiolek M. Brosnan C. F. Rainem C. S. (2007). EAE tolerance induction with Hsp70-peptide complexes depends on H60 and NKG2D activity. *J. Immunol.* 179 4503–4512 17878346 10.4049/jimmunol.179.7.4503

[B21] Gasser S. Orsulic S. Brown E. J. Raulet D. H. (2005). The DNA damage pathway regulates innate immune system ligands for the NKG2D receptor. *Nature* 436 1186–1190 15995699 10.1038/nature03884 PMC1352168

[B22] Groh V. Bruhl A. El-Gabalawy H. Nelson J. L. Spies T. (2003). Stimulation of T cell autoreactivity by anomalous expression of NKG2D and its MIC ligands in rheumatoid arthritis. *Proc. Natl. Acad. Sci. U.S.A.* 100 9452–9457 12878725 10.1073/pnas.1632807100 PMC170939

[B23] Hamerman J. A. Ogasawara K. Lanier L. L. (2004). Toll-like receptor signaling in macrophages induces ligands for the NKG2D receptor. *J. Immunol.* 172 2001–2005 14764662 10.4049/jimmunol.172.4.2001

[B24] He J. Choe S. Walker R. Di Marzio P. Morgan D. O. Landau N. R. (1995). Human immunodeficiency virus type 1 viral protein R (Vpr) arrests cells in the G2 phase of the cell cycle by inhibiting p34cdc2 activity. *J. Virol.* 69 6705–6711 7474080 10.1128/jvi.69.11.6705-6711.1995 PMC189580

[B25] Jinushi M. Takehara T. Kanto T. Tatsumi T. Groh V. Spies T. (2003a). Critical role of MHC class I-related chain A and B expression on IFN-alpha-stimulated dendritic cells in NK cell activation: impairment in chronic hepatitis C virus infection. *J. Immunol.* 170 1249–1256 12538683 10.4049/jimmunol.170.3.1249

[B26] Jinushi M. Takehara T. Tatsumi T. Kanto T. Groh V. Spies T. (2003b). Autocrine/paracrine IL-15 that is required for type I IFN-mediated dendritic cell expression of MHC class I-related chain A and B is impaired in hepatitis C virus infection. *J. Immunol.* 171 5423–5429 14607946 10.4049/jimmunol.171.10.5423

[B27] Jowett J. B. Planelles V. Poon B. Shah N. P. Chen M. L. Chen I. S. (1995). The human immunodeficiency virus type 1 vpr gene arrests infected T cells in the G2 M phase of the cell cycle. *J. Virol.* 69 6304–6313 7666531 10.1128/jvi.69.10.6304-6313.1995 PMC189529

[B28] Kloss M. Decker P. Baltz K. M. Baessler T. Jung G. Rammensee H. G. (2008). Interaction of monocytes with NK cells upon Toll-like receptor-induced expression of the NKG2D ligand MICA. *J. Immunol.* 181 6711–6719 18981088 10.4049/jimmunol.181.10.6711

[B29] Lang P. A. Lang K. S. Xu H. C. Grusdat M. Parish I. A. Recher M. (2012). Natural killer cell activation enhances immune pathology and promotes chronic infection by limiting CD8^+^ T-cell immunity. *Proc. Natl. Acad. Sci. U.S.A.* 109 1210–1215 22167808 10.1073/pnas.1118834109 PMC3268324

[B30] Lanier L. L. (2008). Evolutionary struggles between NK cells and viruses. *Nat. Rev. Immunol.* 8 259–268 18340344 10.1038/nri2276 PMC2584366

[B31] Leavenworth J. W. Wang X. Wenander C. S. Spee P. Cantor H. (2011). Mobilization of natural killer cells inhibits development of collagen-induced arthritis. *Proc. Natl. Acad. Sci. U.S.A.* 108 14584–14589 21873193 10.1073/pnas.1112188108 PMC3167502

[B32] Lee I. F. Qin H. Trudeau J. Dutz J. Tan R. (2004). Regulation of autoimmune diabetes by complete Freund’s adjuvant is mediated by NK cells. *J. Immunol.* 172 937–942 14707066 10.4049/jimmunol.172.2.937

[B33] Li J. Rabinovich B. A. Hurren R. Cosman D. Miller R. G. (2005). Survival versus neglect: redefining thymocyte subsets based on expression of NKG2D ligand(s) and MHC class I. *Eur. J. Immunol.* 35 439–448 15682455 10.1002/eji.200425621

[B34] Lu L. Ikizawa K. Hu D. Werneck M. B. Wucherpfennig K. W. Cantor H. (2007). Regulation of activated CD4^+^ T cells by NK cells via the Qa-1-NKG2A inhibitory pathway. *Immunity* 26 593–604 17509909 10.1016/j.immuni.2007.03.017 PMC3428267

[B35] Lünemann A. Lünemann J. D. Roberts S. Messmer B. Barreira da Silva R. Raine C. S. (2008). Human NK cells kill resting but not activated microglia via NKG2D- and NKp46-mediated recognition. *J. Immunol.* 181 6170–6177 18941207 10.4049/jimmunol.181.9.6170 PMC2596922

[B36] Lünemann A. Lünemann J. D Münz C. (2009). Regulatory NK-cell functions in inflammation and autoimmunity. *Mol. Med.* 15 352–358 19603102 10.2119/molmed.2009.00035 PMC2710290

[B37] Maasho K. Opoku-Anane J. Marusina A. I. Coligan J. E. Borrego F. (2005). NKG2D is a costimulatory receptor for human naive CD8^+^ T cells. *J. Immunol.* 174 4480–4484 15814668 10.4049/jimmunol.174.8.4480

[B38] Matloubian M. Suresh M. Glass A. Galvan M. Chow K. Whitmire J. K. (1999). A role for perforin in downregulating T-cell responses during chronic viral infection. *J. Virol.* 73 2527–2536 9971838 10.1128/jvi.73.3.2527-2536.1999 PMC104500

[B39] Matsumoto Y. Kohyama K. Aikawa Y. Shin T. Kawazoe Y. Suzuki Y. (1998). Role of natural killer cells and TCR gamma delta T cells in acute autoimmune encephalomyelitis. *Eur. J. Immunol.* 28 1681–1688 9603475 10.1002/(SICI)1521-4141(199805)28:05<1681::AID-IMMU1681>3.0.CO;2-T

[B40] Matusali G. Potestà M. Santoni A. Cerboni C. Doria M. (2012). The human immunodeficiency virus type 1 Nef and Vpu proteins downregulate the natural killer cell-activating ligand PVR. *J. Virol.* 86 4496–4504 22301152 10.1128/JVI.05788-11 PMC3318642

[B41] Molinero L. L. Fuertes M. B. Rabinovich G. A. Fainboim L. Zwirner N. W. (2002). Activation-induced expression of MICA on T lymphocytes involves engagement of CD3 and CD28. *J. Leukoc. Biol.* 71 791–797 11994503

[B42] Molinero L. L. Fuertes M. B. Girart M. V. Fainboim L. Rabinovich G. A. Costas M. A. (2004). NFκ B regulates expression of the MHC class I-related chain A gene in activated T lymphocytes. *J. Immunol.* 173 5583–5590 15494508 10.4049/jimmunol.173.9.5583

[B43] Nedvetzki S. Sowinski S. Eagle R. A. Harris J. Vély F. Pende D. (2007). Reciprocal regulation of human natural killer cells and macrophages associated with distinct immune synapses. *Blood* 109 3776–3785 17218381 10.1182/blood-2006-10-052977

[B44] Nielsen N. Ødum N. Ursø B. Lanier L. L. Spee P. (2012). Cytotoxicity of CD56^bright^ NK cells towards autologous activated CD4^+^ T cells is mediated through NKG2D, LFA-1 and TRAIL and dampened via CD94/NKG2A. *PLoS ONE* 7:e31959 10.1371/journal.pone.0031959 PMC3284517 22384114

[B45] Nilsson N. Bremell T. Tarkowski A. Carlsten H. (1999). Protective role of NK1.1+ cells in experimental *Staphylococcus aureus* arthritis. *Clin. Exp. Immunol.* 117 63–69 10403917 10.1046/j.1365-2249.1999.00922.x PMC1905466

[B46] Nolte-’t Hoen E. N. M. Almeida C. R. Cohen N. R. Nedvetzki S. Yarwood H. (2007). Increased surveillance of cells in mitosis by human NK cells suggests a novel strategy for limiting tumor growth and viral replication. *Blood* 109 670–673 16960147 10.1182/blood-2006-07-036509

[B47] Norman J. M. Mashiba M. McNamara L. A. Onafuwa-Nuga A. Chiari-Fort E. Shen W. (2011). The antiviral factor APOBEC3G enhances the recognition of HIV-infected primary T cells by natural killer cells. *Nat. Immunol.* 12 975–983 21874023 10.1038/ni.2087 PMC3530928

[B48] Noval Rivas M. Hazzan M. Weatherly K. Gaudray F. Salmon I. Braun M. Y. (2010). NK cell regulation of CD4 T cell-mediated graft-versus-host disease. *J. Immunol.* 184 6790–6798 20488796 10.4049/jimmunol.0902598

[B49] Nowbakht P. Ionescu M. C. Rohner A. Kalberer C. P. Rossy E. Mori L. (2005). Ligands for natural killer cell-activating receptors are expressed upon the maturation of normal myelomonocytic cells but at low levels in acute myeloid leukemias. *Blood* 105 3615–3622 15657183 10.1182/blood-2004-07-2585

[B50] Ogasawara K. Benjamin J. Takaki R. Phillips J. H. Lanier L. L. (2005). Function of NKG2D in natural killer cell-mediated rejection of mouse bone marrow grafts. *Nat. Immunol.* 6 938–945 16086018 10.1038/ni1236 PMC1351289

[B51] Olson J. A. Zeiser R. Beilhack A. Goldman J. J. Negrin R. S. (2009). Tissue-specific homing and expansion of donor NK cells in allogeneic bone marrow transplantation. *J. Immunol.* 183 3219–3228 19657090 10.4049/jimmunol.0804268 PMC2880476

[B52] Olson J. A. Leveson-Gowere D. B. Baker J. Beilhack A. Negrin R. S. (2010). NK cells mediate reduction of GVHD by inhibiting activated, aloreactive T cells while retaining GVT effects. *Blood* 115 4293–4301 20233969 10.1182/blood-2009-05-222190 PMC2879101

[B53] Oricchio E. Saladino C. Iacovelli S. Soddu S. Cundari E. (2006). ATM is activated by default in mitosis, localizes at centrosomes and monitors mitotic spindle integrity. *Cell Cycle* 5 88–92 16319535 10.4161/cc.5.1.2269

[B54] Pende D. Castriconi R. Romagnani P. Spaggiari G. M. Marcenaro S. Dondero A. (2006). Expression of the DNAM-1 ligands, Nectin-2 (CD112) and poliovirus receptor (CD155), on dendritic cells: relevance for natural killer-dendritic cell interaction. *Blood* 107 2030–2036 16304049 10.1182/blood-2005-07-2696

[B55] Pham T. N. Richard J. Gerard F. C. Power C. Cohen E. A. (2011). Modulation of NKG2D-mediated cytotoxic functions of natural killer cells by viral protein R from HIV-1 primary isolates. *J. Virol.* 85 12254–12261 21957298 10.1128/JVI.05835-11 PMC3209390

[B56] Poggi A. Prevosto C. Massaro A. M. Negrini S. Urbani S. Pierri I. (2005). Interaction between human NK cells and bone marrow stromal cells induces NK cell triggering: role of NKp30 and NKG2D receptors. *J. Immunol.* 175 6352–6360 16272287 10.4049/jimmunol.175.10.6352

[B57] Qiao Y. Liu B. Li Z. (2008). Activation of NK cells by extracellular heat shock protein 70 through induction of NKG2D ligands on dendritic cells. *Cancer Immun.* 8 12–20 18613644 PMC2935777

[B58] Rabinovich B. A. Li J. Shannon J. Hurren R. Chalupny J. Cosman D. (2003). Activated, but not resting, T cells can be recognized and killed by syngeneic NK cells. *J. Immunol.* 170 3572–3576 12646619 10.4049/jimmunol.170.7.3572

[B59] Re F. Braaten D. Frankem E. K. Luban J. (1995). Human immunodeficiency virus type 1 Vpr arrests the cell cycle in G2 by inhibiting the activation of p34cdc2-cyclin B. *J. Virol.* 69 6859–6864 7474100 10.1128/jvi.69.11.6859-6864.1995 PMC189600

[B60] Richard J. Sindhu S. Pham T. N. Belzile J. P. Cohen E. A. (2010). HIV-1 Vpr up-regulates expression of ligands for the activating NKG2D receptor and promotes NK cell-mediated killing. *Blood* 115 1354–1363 20008788 10.1182/blood-2009-08-237370 PMC3955183

[B61] Roshal M. Kim B. Zhu Y. Nghiem P. Planelles V. (2003). Activation of the ATR-mediated DNA damage response by the HIV-1 viral protein R. *J. Biol. Chem.* 278 25879–25886 12738771 10.1074/jbc.M303948200

[B62] Rossini G. Cerboni C. Santoni A. Landini M. P. Landolfo S. Gatti D. (2012). Interplay between human cytomegalovirus and intrinsic/innate host responses: a complex bidirectional relationship. *Mediat. Inflamm.* 2012 1–16 10.1155/2012/607276 PMC3371353 22701276

[B63] Roy S. Barnes P. F. Garg A. Wu S. Cosman D. Vankayalapati R. (2008). NK cells lyse T regulatory cells that expand in response to an intracellular pathogen. *J. Immunol.* 180 1729–1736 18209070 10.4049/jimmunol.180.3.1729

[B64] Ruggeri L. Capanni M. Urbani E. Perruccio K. Shlomchik W. D. Tosti A. (2002). Effectiveness of donor natural killer cell alloreactivity in mismatched hematopoietic transplants. *Science* 295 2097–2100 11896281 10.1126/science.1068440

[B65] Schott E. Bonasio R. Ploegh H. L. (2003). Elimination of *in vivo* developing T cells by natural killer cells. *J. Exp. Med.* 198 1213–1224 14568980 10.1084/jem.20030918 PMC2194238

[B66] Schrama D. Terheyden P. Otto K. Kämmerer U. Bröcker E. B. Lühder F. (2006). Expression of the NKG2D ligand UL16 binding protein-1 (ULBP-1) on dendritic cells. *Eur. J. Immunol.* 36 65–72 16342232 10.1002/eji.200535115

[B67] Schulz U. Kreutz M. Multhoff G. Stoelcker B. Köhler M. Andreesen R. (2010). Interleukin-10 promotes NK cell killing of autologous macrophages by stimulating expression of NKG2D ligands. *Scand. J. Immunol.* 72 319–331 20883317 10.1111/j.1365-3083.2010.02435.x

[B68] Shi F. D. Takeda K. Akira S. Sarvetnick N. Ljunggren H. G. (2000). IL-18 directs autoreactive T cells and promotes autodestruction in the central nervous system via induction of IFN-gamma by NK cells. *J. Immunol.* 165 3099–3104 10975822 10.4049/jimmunol.165.6.3099

[B69] Shi F. D Van Kaer L. (2006). Reciprocal regulation between natural killer cells and autoreactive T cells. *Nat. Rev. Immunol.* 6 751–760 16998508 10.1038/nri1935

[B70] Shibuya A. Campbell D. Hannum C. Yssel H. Franz-Bacon K. McClanahan T. (1996). DNAM-1, a novel adhesion molecule involved in the cytolytic function of T lymphocytes. *Immunity* 4 573–581 8673704 10.1016/s1074-7613(00)70060-4

[B71] Sivori S. Carlomagno S. Falco M. Romeo E. Moretta L. Moretta A. (2011). Natural killer cells expressing the KIR2DS1-activating receptor efficiently kill T-cell blasts and dendritic cells: implications in haploidentical HSCT. *Blood* 117 4284–4292 21355085 10.1182/blood-2010-10-316125

[B72] Skov S. Pedersen M. T. Andresen L. Straten P. T. Woetmann A. Odum N. (2005). Cancer cells become susceptible to natural killer cell killing after exposure to histone deacetylase inhibitors due to glycogen synthase kinase-3-dependent expression of MHC class I-related chain A and B. *Cancer Res.* 65 11136–11145 16322264 10.1158/0008-5472.CAN-05-0599

[B73] Smeltz R. B. Wauben M. H. Wolf N. A. Swanborg R. H. (1999). Critical requirement for aspartic acid at position 82 of myelin basic protein 73-86 for recruitment of V beta 8.2+ T cells and encephalitogenicity in the Lewis rat. *J. Immunol.* 162 829–836 9916705

[B74] Spaggiari G. M. Capobianco A. Becchetti S. Mingari M. C. Moretta L. (2006). Mesenchymal stem cell-natural killer cell interactions: evidence that activated NK cells are capable of killing MSCs, whereas MSCs can inhibit IL-2-induced NK-cell proliferation. *Blood* 107 1484–1490 16239427 10.1182/blood-2005-07-2775

[B75] Soriani A. Zingoni A. Cerboni C. Iannitto M. L. Ricciardi M. R. Di Gialleonardo V. (2009). ATM-ATR dependent up-regulation of DNAM-1 and NKG2D ligands on multiple myeloma cells by therapeutic agents results in enhanced NK-cell susceptibility and is associated with a senescent phenotype. *Blood* 113 3503–3511 19098271 10.1182/blood-2008-08-173914

[B76] Stanietsky N. Simic H. Arapovic J. Toporik A. Levy O. Novik A. (2009). The interaction of TIGIT with PVR and PVRL2 inhibits human NK cell cytotoxicity. *Proc. Natl. Acad. Sci. U.S.A.* 106 17858–17863 19815499 10.1073/pnas.0903474106 PMC2764881

[B77] Su H. C. Nguyen K. B. Salazar-Mather T. P. Ruzek M. C. Dalod M. Y. Biron C. A. (2001). NK cell functions restrain T cell responses during viral infections. *Eur. J. Immunol.* 31 3048–3055 11592081 10.1002/1521-4141(2001010)31:10<3048::aid-immu3048>3.0.co;2-1

[B78] Tahara-Hanaoka S. Shibuya K. Onoda Y. Zhang H. Yamazaki S. Miyamoto A. (2004). Functional characterization of DNAM-1 (CD226) interaction with its ligands PVR (CD155) and nectin-2 (PRR-2/CD112). *Int. Immunol.* 16 533–538 15039383 10.1093/intimm/dxh059

[B79] Tahara-Hanaoka S. Shibuya K. Kai H. Miyamoto A. Morikawa Y. Ohkochi N. (2006). Tumor rejection by the poliovirus receptor family ligands of the DNAM-1 (CD226) receptor. *Blood* 107 1491–1496 16249389 10.1182/blood-2005-04-1684

[B80] Tanaka T. Kajstura M. Halicka H. D. Traganos F. Darzynkiewicz Z. (2007). Constitutive histone H2AX phosphorylation and ATM activation are strongly amplified during mitogenic stimulation of lymphocytes. *Cell Prolif.* 40 1–13 17227291 10.1111/j.1365-2184.2007.00417.x PMC3860878

[B81] Van Belle T. L von Herrath M. G. (2009). The role of the activating receptor NKG2D in autoimmunity. *Mol. Immunol.* 47 8–11 19286259 10.1016/j.molimm.2009.02.023

[B82] Venkataraman G. M. Suciu D. Groh V. Boss J. M. Spies T. (2007). Promoter region architecture and transcriptional regulation of the genes for the MHC class I-related chain A and B ligands of NKG2D. *J. Immunol.* 178 961–969 17202358 10.4049/jimmunol.178.2.961

[B83] Waggoner S. N. Cornberg M. Selin L. K. Welsh R. M. (2011). Natural killer cells act as rheostats modulating antiviral T cells. *Nature* 481 394–398 22101430 10.1038/nature10624 PMC3539796

[B84] Ward J. Bonaparte M. Sacks J. Guterman J. Fogli M. Mavilio D. (2007). HIV modulates the expression of ligands important in triggering natural killer cell cytotoxic responses on infected primary T-cell blasts. *Blood* 110 1207–1214 17513617 10.1182/blood-2006-06-028175 PMC1939902

[B85] Ward J. Davis Z. DeHart J. Zimmerman E. Bosque A. Brunetta E. (2009). HIV-1 Vpr triggers natural killer cell-mediated lysis of infected cells through activation of the ATR-mediated DNA damage response. *PLoS Pathog.* 5:10 10.1371/journal.ppat.1000613 PMC2747015 19798433

[B86] Xu W. Fazekas G. Hara H. Tabira T. (2005). Mechanism of natural killer (NK) cell regulatory role in experimental autoimmune encephalomyelitis. *J. Neuroimmunol.* 163 24–30 15885305 10.1016/j.jneuroim.2005.02.011

[B87] Yamaji O. Nagaishi T. Totsuka T. Onizawa M. Suzuki M. Tsuge N. (2012). The development of colitogenic CD4(+) T cells is regulated by IL-7 in collaboration with NK cell function in a murine model of colitis. *J. Immunol.* 188 2524–2536 22331065 10.4049/jimmunol.1100371

[B88] Yang C. Kastan M. (2000). Participation of ATM in insulin signalling through phosphorylation of eIF-4E-binding protein 1. *Nat. Cell Biol.* 2 893–898 11146653 10.1038/35046542

[B89] Zhang B. Yamamura T. Kondo T. Fujiwara M. Tabira T. (1997). Regulation of experimental autoimmune encephalomyelitis by natural killer (NK) cells. *J. Exp. Med.* 186 1677–1687 9362528 10.1084/jem.186.10.1677 PMC2199138

